# Polarized cell migration induces cancer type-specific CD133/integrin/Src/Akt/GSK3β/β-catenin signaling required for maintenance of cancer stem cell properties

**DOI:** 10.18632/oncotarget.5703

**Published:** 2015-10-19

**Authors:** Ying-Jhen Su, Wei-Hsin Lin, Yi-Wen Chang, Kuo-Chen Wei, Chi-Lung Liang, Shin-Cheh Chen, Jia-Lin Lee

**Affiliations:** ^1^ Institute of Molecular and Cellular Biology, National Tsing Hua University, Hsinchu, Taiwan; ^2^ Department of Orthopedics, National Taiwan University Hospital Hsin-Chu Branch, Hsinchu, Taiwan; ^3^ Department of Neurosurgery, Chang-Gung Memorial Hospital, Linkou, Taiwan; ^4^ Department of Surgery, Chang-Gung Memorial Hospital, Linkou, Taiwan; ^5^ Department of Medical Science, National Tsing Hua University, Hsinchu, Taiwan

**Keywords:** β-catenin, cancer stem cell, CD133, cell surface marker, extracellular matrix

## Abstract

CD133 is widely used as a surface marker to isolate cancer stem cells (CSCs). Here we show that in CSCs CD133 contributes to β-catenin-mediated transcriptional activation and to the self-renewal capacity of sphere-forming and side-population (SP) cells in cell lines from brain, colon and lung cancers, but not gastric or breast cancers. In chromatin immunoprecipitation assays, β-catenin binding to the proximal promoter regions of *ITGA2-4* and *ITGA10-11* in brain, colon and lung cancer cell lines could be triggered by CD133, and β-catenin also bound to the proximal promoter regions of *ITGB6* and *ITGB8* in cell lines from gastric and breast cancers. CD133 thus induces β-catenin binding and transcriptional activation of diverse targets that are cancer type-specific. Cell migration triggered by wounding CD133^+^ cells cultured on ECM-coated dishes can induce polarity and lipid raft coalescence, enhancing CD133/integrin signaling and asymmetric cell division. In response to directional cues, integrins, Src and the Par complex were enriched in lipid rafts, and the assembly and activation of an integrated CD133-integrin-Par signaling complex was followed by Src/Akt/GSK3β signaling. The subsequent increase and nuclear translocation of β-catenin may be a regulatory switch to increase drug resistance and stemness properties. Collectively, these findings 1) indicate that a polarized cell migration-induced CD133/integrin/Src/Akt/GSK3β/β-catenin axis is required for maintenance of CSC properties, 2) establish a function for CD133 and 3) support the rationale for targeting CD133 in cancer treatment.

## INTRODUCTION

Promonin-1, also called CD133, is a five-transmembrane glycoprotein that typically localizes at membrane protrusions [1]. CD133 was first discovered as a surface antigen on CD34^+^ hematopoietic stem cells [2] and has been used in combination with other cell-surface markers, including CD24, CD34, and CD44, to isolate cancer stem cells (CSCs) from a variety of tumors [3]. The precise functions of CD133 are not yet known. Its unique distribution suggests CD133 may be involved in membrane organization. This idea is supported by the fact that loss of CD133 from the plasma membrane of human retinal cells causes retinal degeneration, possibly due to impaired generation of evaginations and/or impaired conversion of evaginations to disks [4]. Topologically, CD133 is located in cholesterol-containing lipid rafts in membrane microdomains, where it is involved in mediating signaling cascades [5]. CD133 may also affect whether cell division is symmetric or asymmetric [6] and may be a mediator of cellular polarity and migration [7]. The function of CD133 is even less clear in the context of cancer. Despite its ubiquitous presence on CSCs from various solid tumors, it is unknown whether the intracellular signaling downstream of CD133 contributes to the maintenance of cellular stemness. CD133 signaling reportedly enhances self-renewal and tumorigenic potential in glioma stem cells through the Src and phosphatidylinositol 3-kinase (PI3K)/Akt pathways [8, 9]. Clinically, strong CD133 expression correlates with chemo/radio-resistance and a poor prognosis [10].

Cellular polarity, or the asymmetric distribution of a cell's components, is a fundamental feature of the epithelium. Establishment of proper apical-basal polarity is crucial to the shape, organization and function of epithelial tissue [11]. Loss of polarity is associated with disruption of cellular architecture, loss of intercellular adhesion and contact inhibition, and eventually cancer initiation and progression [12]. Partition-defective 6 (Par6) is a critical regulator of apical-basal polarity, polarized cell movement and asymmetric cell division (ACD). The so-called Par polarity complex is composed of Par6, Par3, atypical protein kinase C (aPKC) and cell division control protein 42. The complex is recruited by signals mediated by integrin and TGFβ [13]. ACD is a feature of stem and progenitor cells, and several key regulators of ACD are known tumor suppressors; thus loss of cellular polarity and dysregulation of ACD may lead to tumor formation, invasion and metastasis [14]. CSCs retain the ability to divide asymmetrically [15], which accounts, at least in part, for the heterogeneity seen in most tumors and suggests that ACD may also contribute to the maintenance of CSCs. The exact mechanism(s) by which cellular polarity and ACD affects the properties of CSCs is not known, however.

Our findings in the present study suggest there are links among the tumor niche, CD133, cell polarity, ACD and CSCs, and indicate that targeting CD133 may be an effective means of interfering with tumorigenesis and/or metastasis.

## RESULTS

### CD133 levels are directly associated with tumor stage in lung cancer but not breast cancer

**Figure 1:**
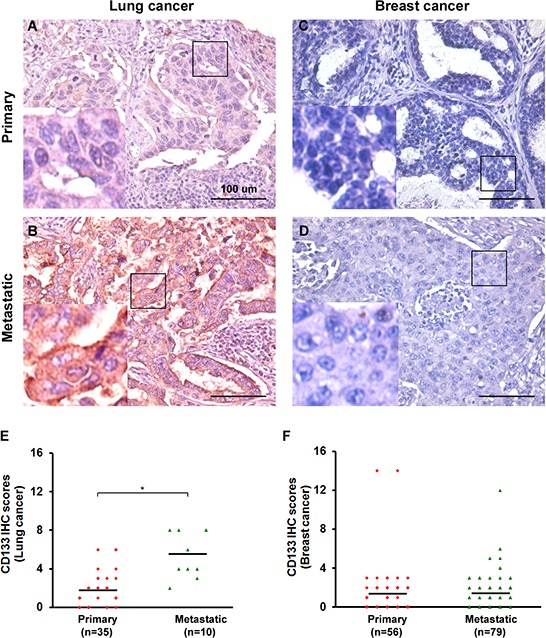
CD133 is clinically significant in lung cancer but not breast cancer patients **A–D.** IHC for CD133 in representative tumor tissues from lung (in A-B) and breast (in C-D) cancer patients. Both primary and metastatic cancer specimens are shown. Tissues were counterstained in hematoxylin. Bars, 100 μm. Left bottom in each panel shows enlarged images of the boxed areas. **E–F.** Scatter plots show the distribution of IHC intensity scores of various proteins among lung (35 primary and 10 metastatic; in E) and breast (56 primary and 79 metastatic; in F) cancer specimens. IHC scores = % of positive cells × staining intensity. Black bars indicate the average score within each group of specimens. *P < 0.05.

To validate the clinical relevance of CD133 to human cancers and its contribution to maintenance of the CSC population in tumors, we used immunohistochemical (IHC) staining and scoring analysis [16, 17] to assess CD133 in sections of human lung (35 primary and 10 metastatic) and breast (56 primary and 79 metastatic) cancer. IHC scoring was determined by multiplying the staining intensity by the percentage of positive tumor cells [16, 17]. We found that CD133 levels were higher in metastatic lung cancers than primary cancers (Figure 1A–1B, 1E), whereas there were no differences between metastatic and primary breast cancers (Figure 1C–1D, 1F). There was also a direct association between CD133 levels and tumor stage in lung cancer, which reached a maximum in metastatic lung tumors. This association was not seen in breast cancer.

### CD133 is a functionally important cell-surface marker in CSCs

**Figure 2:**
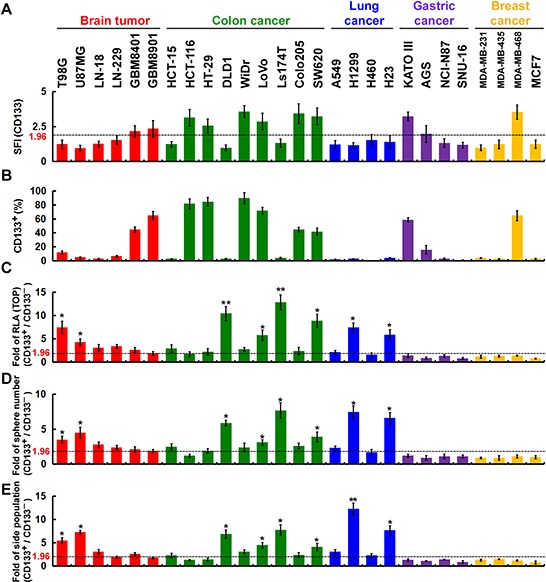
The cell-surface marker CD133 is functionally important in CSCs **A** and **B.** Cells were incubated with isotype IgG (control) or anti-CD133 and then labeled with Alexa Fluor 488-conjugated secondary antibody. Fluorescence intensity was determined using flow cytometry. The specific fluorescence index (SFI) was calculated as the ratio of the mean fluorescence obtained with anti-CD133 to that with isotype IgG (in A). The percentage of CD133^+^ cells was determined using flow cytometry (in B). **C.** Cells were fractionated using FACS into CD133^+^ and CD133^−^ fractions, and the CD133^+^/CD133^−^ ratio of the TOPflash luciferase reporter activity was calculated. RLA, relative luciferase activity. **D.** *In vitro* quantification of spheres formed by cells during four serial passages. The spheres were cultured for 12 days, and those with diameters > 30 μm were counted. Each sphere was derived from single cells. The CD133^+^/CD133^−^ ratio of spheres formed over four serial passages was counted. **E.** Cells were stained with Hoechst 33342, and the CD133^+^/CD133^−^ ratio of SP cells was counted. Data were derived from three independent experiments and are presented as the mean ± SD. *P < 0.05; **P < 0.01 (*t*-test).

Although cancer cell lines are initially established from a single-cell clone, they likely become heterogeneous after long-term culture owing to the genetic instability of cancer cells. This makes it possible to isolate fractions with different characteristics, particularly the CSC population, for which there are specific cell surface markers, including CD133. CD133 levels (Figure 2A) and the size of the CD133^+^ fraction (Figure 2B) in various cancer cell lines were determined using flow cytometry. We first used fluorescence-activated cell sorting (FACS) to divide the cancer cells into CD133^+^ and CD133^−^ fractions. There appears to be a link between CD133 and the Wnt/β-catenin pathway [18, 19], which CD133 can stabilize, leading to activation of β-catenin signaling targets. In the present study, TOPflash reporter activity (to measure β-catenin-dependent transcriptional activity) in the CD133^+^ fraction was increased 5- to 10-fold compared to the CD133^−^ fraction in some cell lines from brain, colon and lung cancers, but not gastric or breast cancers (Figure 2C). In addition, CD133 enhanced the self-renewal capability of the sphere-forming and side-population (SP) cells. Self-renewal capability of the sphere-forming cells (Figure 2D) over four serial passages and SP cells (Figure 2E) were increased exclusively in some CD133^+^ cell lines from brain, colon and lung cancers. Taken together, these findings suggest the cell-surface marker CD133 is functionally important for β-catenin-mediated transcriptional activation in CSCs and for the self-renewal capability of the sphere-forming and SP cells in some cell lines from brain, colon and lung cancers, but not gastric or breast cancers.

### CD133 intracellular domains promote β-catenin-mediated transcriptional activity and maintenance of CSC properties

**Figure 3:**
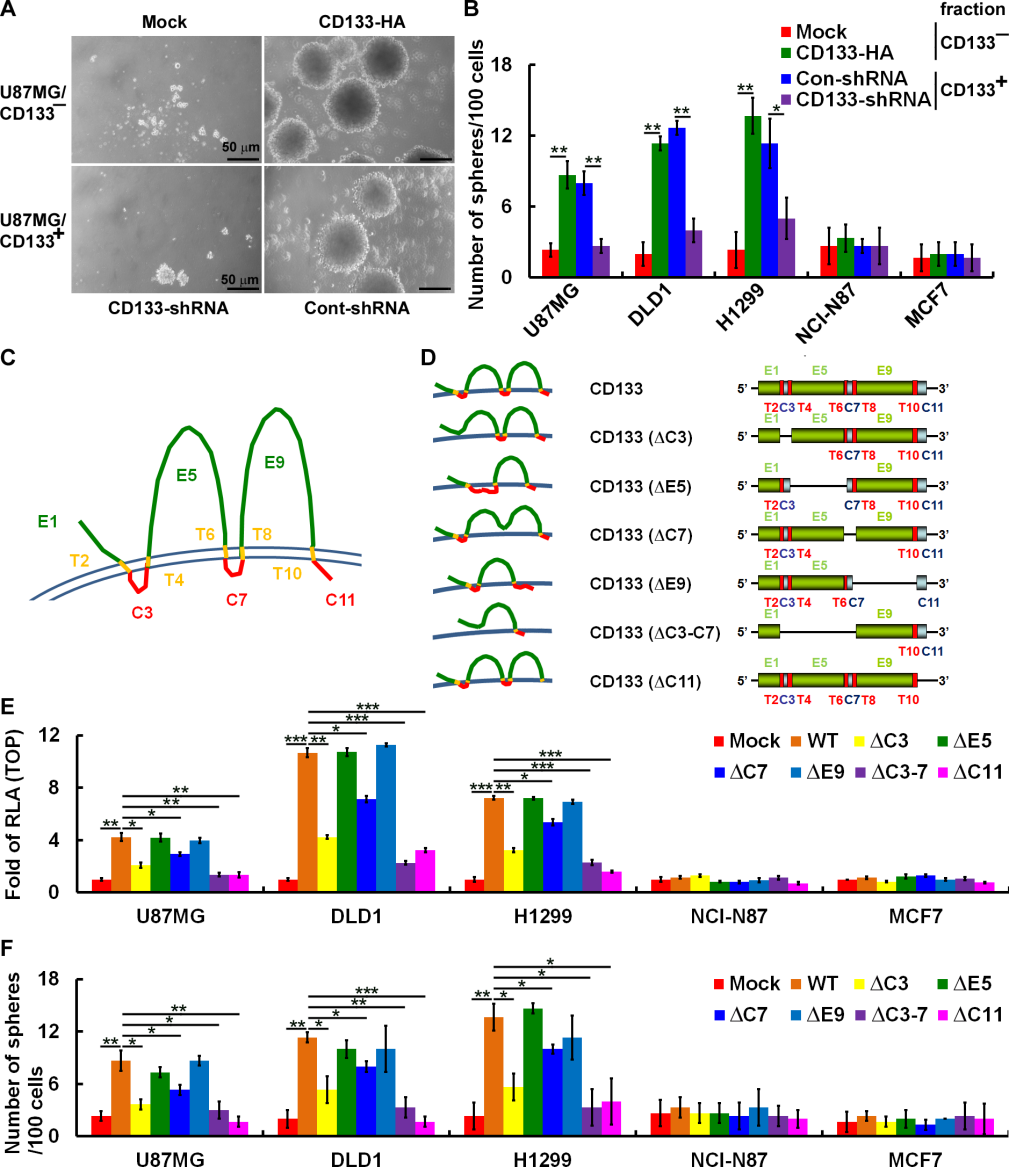
CD133 intracellular domains are required to enhance β-catenin-mediated transcription and maintain CSC properties **A.** Sphere formation by U87MG cells was assessed, and microscopic analysis of spheres cultivated in suspension for 12 days was carried out. Top panel: U87MG/CD133^−^ cells were transfected with a plasmid encoding CD133 (CD133-HA) or control vector (Mock); bottom panel: U87MG/CD133^+^ cells were infected with lentivirus encoding shRNA targeting CD133 (CD133-shRNA) or control scrambled shRNA (Cont-shRNA). Bars, 50 μm. **B.** *In vitro* quantification of spheres formed by cells described in A during four serial passages. **C** and **D.** Schematic representation of wild-type and extracellular (E, green) and intracellular (C, red) domain-deleted CD133 mutants. T, transmembrane domain, yellow. **E.** Whole-cell lysates were prepared from CD133^−^ cells transfected with plasmids encoding wild-type (WT) and E and C domain-deleted CD133. A TOP/FOPflash luciferase reporter assay was performed. **F.** *In vitro* quantification of spheres over four serial passages formed by CD133^−^ stable clones expressing CD133 as in E. Data in B, E and F were derived from three independent experiments and are presented as the mean ± S.D. *P < 0.05; **P < 0.01; ***P < 0.005 (*t*-test).

We established cell clones expressing various CD133 mutants or control vectors in CD133^−^ cells (sorted from brain U87MG, colon DLD1, lung H1299, gastric NCI-N87 and breast MCF7 cells using FACS). After culture for 12 days under sphere-forming conditions, U87MG/CD133^−^/CD133-HA (ectopic expression of CD133) and U87MG/CD133^+^ (endogenous expression of CD133) cells produced sphere colonies, while U87MG/CD133^−^/Mock cells did not (Figure 3A). Consistent with this observation, elimination of *CD133* transcripts in U87MG/CD133^+^ cells using lentivirus-based RNA interference inhibited sphere formation. When we test the self-renewal capability of the sphere-forming cells, we found that ~12 (CD133-HA expressed in CD133^−^ U87MG, DLD1 and H1299 cells) and ~10 (control short hairpin RNA [shRNA, Control-shRNA] expressed in CD133^+^ U87MG, DLD1 and H1299 cells) spheres formed per 100 seeded cells (12% and 10%, respectively), whereas < 4% of seeded cells formed spheres among CD133^−^/Mock and CD133^+^/CD133-shRNA cells (Figure 3B). To further define the CD133 domains involved in self-renewal of sphere-forming cells, a set of extracellular- (E) and intracellular- (C) domain deletion mutants were generated from wild-type cells (Figure 3C and 3D). Wild-type CD133 promoted β-catenin-mediated transcriptional activity (Figure 3E) and self-renewal (Figure 3F) in CD133^−^ U87MG, DLD1 and H1299 cells, but CD133ΔC3, CD133ΔC7, CD133ΔC3-7 and CD133ΔC11 did not promote the transcriptional activity (Figure 3E) or self-renewal (Figure 3F).

### CD133-mediated maintenance of CSC properties is depend on cancer-specific integrin/extracellular matrix (ECM) signaling

**Figure 4:**
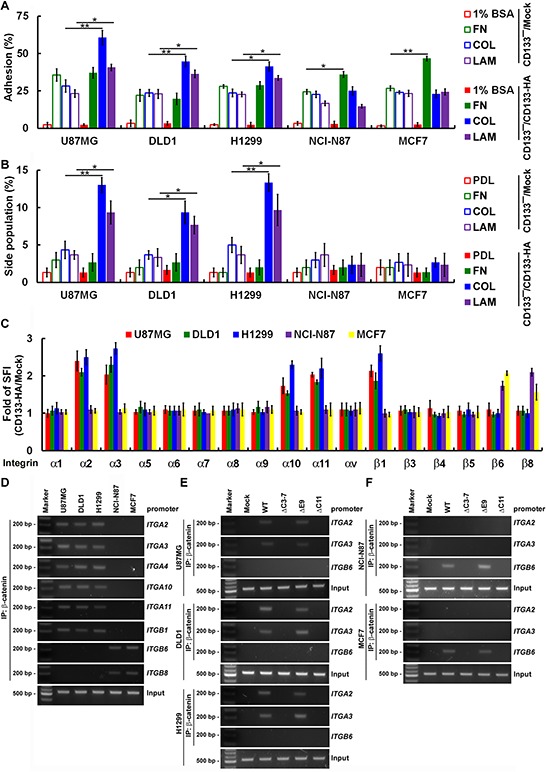
CD133 activity to maintain CSC properties depend on cancer type-specific integrin/ECM signaling **A.** Using FACS, cells were fractionated into CD133^+^ and CD133^−^ fractions. Subconfluent CD133^−^/Mock and CD133^−^/CD133-HA cells were replated on 1% bovine serum albumin (1% BSA)-, fibronectin (FN)-, collagen (COL)- or laminin (LAM)-coated dishes and allowed to adhere for 30 min. **B.** Subconfluent CD133^−^/Mock and CD133^−^/CD133-HA cells were replated on poly-d-lysine (PDL)-, FN-, COL- or LAM-coated dishes and allowed to adhere for 24 h. Cells were stained with Hoechst 33342, and the percentage of SP cells was calculated. **C.** Cells were incubated with isotype IgG (control) or anti-integrin and then labeled with Alexa Fluor 488-conjugated secondary antibody. Fluorescence intensity was determined with flow cytometry. The SFI was calculated as the ratio of the mean fluorescence obtained with anti-integrin to that obtained with isotype IgG. The CD133-HA/Mock SFI ratio was calculated. **D–F.** Nuclear extracts were prepared from CD133^+^ (D) and CD133^−^/CD133-HA (WT or mutants; E-F) cells. For ChIP, the DNA was immunoprecipitated with anti-β-catenin. Extracted DNA was analyzed using PCR with primers spanning the proximal promoter regions of *ITGA2, ITGA3, ITGA4, ITGA10, ITGA11, ITGB1, ITGB6* or *ITGB8*. Data in (A–C) were derived from three independent experiments and are presented as the mean ± S.D. *P < 0.05; **P < 0.01 (*t*-test).

ECM is crucial for maintenance of CSC properties, as indicated by the observation that pure populations of adhesive glioma stem cells can be expanded in laminin-coated culture plates [20]. As shown in Figure 4A, CD133 expression enhanced adherence of U87MG, DLD1 and H1299 cells to collagen/laminin, as well as attachment of NCI-N87 and MCF7 cells to fibronectin. In addition, culturing CD133^−^ and CD133^+^ NCI-N87 and MCF7 on ECM (fibronectin, collagen and laminin)-coated dishes had no effect on SP cell percentages, whereas SP cells percentages were increased in CD133^+^ U87MG, DLD1 and H1299 cells cultured on collagen- or laminin-coated dishes (Figure 4B).

We next used FACS to assess expression of integrin subunits in U87MG, DLD1 and H1299 cells. In the CD133^+^ fraction, levels of collagen receptors (α2, α10 and α11 integrins) and laminin receptors (α3 integrin) were 2- to 3-fold higher than in the CD133^−^ fraction (Figure 4C). Similarly, levels of fibronectin receptors (β6 and β8 integrins) were enhanced in CD133^+^ NCI-N87 and MCF7 cells. These FACS results were validated by ChIP assays (Figure 4D–4F). β-catenin binding to the proximal promoter regions of *ITGA2, ITGA3, ITGA4, ITGA10* and *ITGA11* in U87MG, DLD1 and H1299 cells could be triggered by CD133 (Figure 4D). β-catenin also bound to the proximal promoter regions of *ITGB6* and *ITGB8* in NCI-N87 and MCF7 cells. These results were confirmed by mapping of the CD133 domains affecting β-catenin-mediated transcriptional activity. CD133ΔC3-7 and CD133ΔC11 failed to promote β-catenin binding to the proximal promoter region of integrin genes and the consequent transcriptional activity (Figure 4E and 4F). Taken together, these results show that CD133 elicits β-catenin binding and transcriptional activation of diverse targets that are cancer type-specific.

### Cell migration triggered by wounding is a pivotal step toward inducing polarity and lipid raft coalescence and enhancing CD133/integrin signaling

**Figure 5:**
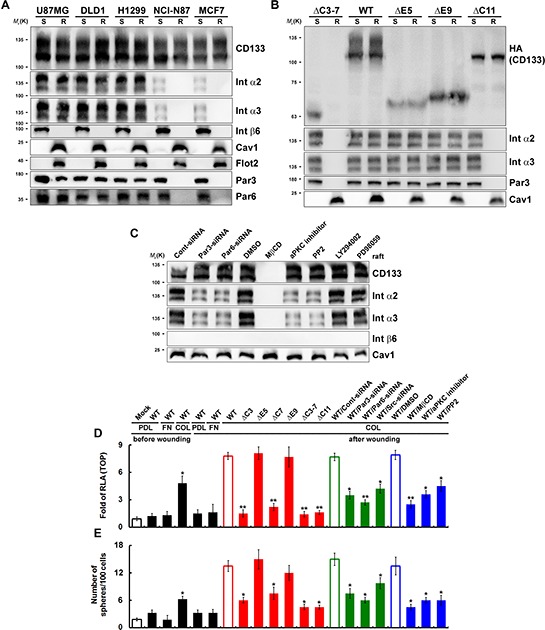
Cell migration triggered by wounding is pivotal for achieving polarity, inducing lipid raft coalescence and activating CD133/integrin signaling **A** and **B.** CD133^+^ (in A) and CD133^−^/CD133-HA (WT or mutants) (in B) cells cultured on collagen-coated dishes were lysed in cold Triton X-100 buffer 24 post-wounding. R: The Triton X-100-insoluble raft fraction; S: The Triton X-100-soluble fraction. Both fractions were analyzed with immunoblotting. **C.** U87MG/CD133^+^ cells cultured on collagen-coated dishes were pretreated with a lipid raft-destabilizing drug (MβCD); an inhibitor of aPKC (Gö6983), Src (PP2), PI3K (LY294002) or MAPK (PD98059); or siRNA targeting Par3 or Par6. R fraction was analyzed by immunoblotting 24 h post-wounding. **D** and **E.** U87MG/CD133^−^/CD133-HA (WT or mutants) cells cultured on poly-d-lysine (PDL)-, fibronectin (FN)- or collagen (COL)-coated dishes were pretreated with inhibitors or siRNA as in C. TOPflash luciferase reporter (in D) and self-renewal capability (the sphere-forming cells) assays (in E) were performed 24 hours post-wounding. RLA, relative luciferase activity. Data in (D–E) were derived from three independent experiments and are presented as the mean ± S.D. *P < 0.05; **P < 0.01 (*t*-test).

Partitioning of molecules into specific membrane microdomains, termed rafts, may localize proteins at the front or the rear of moving cells, enabling rafts to function as platforms for local activation and coordination of the signaling pathways during cell migration [21]. We examined the distribution of CD133/integrins in lipid rafts after inducing cell (cultured on collagen-coated dishes) migration by wounding (establishing the polarity in migrating cells [22]). After solubilizing the CD133^+^ cells in cold Triton X-100 buffer and subsequent sucrose gradient centrifugation [23], the lipid rafts were recovered from low-density fractions 2–4, as indicated by the presence of caveolin-1 and flotillin-2 [23, 24]. The Triton X-100-soluble cellular components were distributed over fractions 7–10. Cell migration triggered by wounding induced lipid raft coalescence. It also promoted the enrichment of CD133, Par3, Par6 and integrins (α2 and α3 integrins were induced but not β6 integrin) in the lipid rafts in U87MG, DLD1 and H1299 cells, but not NCI-N87 and MCF7 cells (Figure 5A). After wounding, two CD133 mutants, CD133ΔC3-7 (defective association with lipid rafts) and CD133ΔC11 (defective association with Src; Src kinase interacts with and phosphorylates the cytoplasmic domain of CD133 [8, 25]) did not show lipid raft coalescence (Figure 5B). Of the inhibitors tested, a lipid raft-destabilizing drug (MβCD), an aPKC inhibitor (Gö6983), a Src inhibitor (PP2), and siRNA targeting Par3 and Par6 (perturbed lipid raft coalescence and cell polarization) decreased localization of integrins (α2 and α3 integrins) into the raft fractions (Figure 5C). By contrast, neither PI3K (LY294002) nor MAPK (PD98059) inhibitors had any effect.

To determine whether the formation of polarized CD133/integrin, and the resultant lipid raft coalescence, is required for β-catenin-dependent transcriptional activity and the CSC phenotype, we evaluated the response to wounding by cells expressing a CD133 mutant or were pretreated with an inhibitor. After wounding, wild-type CD133^+^ cells cultured on collagen-coated dishes showed enhanced TOPflash reporter activity (Figure 5D) and self-renewal of sphere-forming cells (Figure 5E). Pretreatment with inhibitors or mutants that disrupt CD133/integrin polarization (siRNA against Par3 and Par6) and lipid raft coalescence [CD133ΔC3, CD133ΔC7, CD133ΔC3-7, and CD133ΔC11 mutants; a lipid raft-destabilizing drug (MβCD); siRNA against Src; an aPKC inhibitor (Gö6983), and a Src inhibitor (PP2)] abolished CD133-mediated β-catenin-dependent transcriptional activity (Figure 5D) and the CSC phenotype (Figure 5E). These results indicate that cell migration triggered by wounding can induce polarity and lipid raft coalescence and enhance CD133/integrin signaling.

### Polarized localization of CD133/integrins in migrating cells contributes to ACD

**Figure 6:**
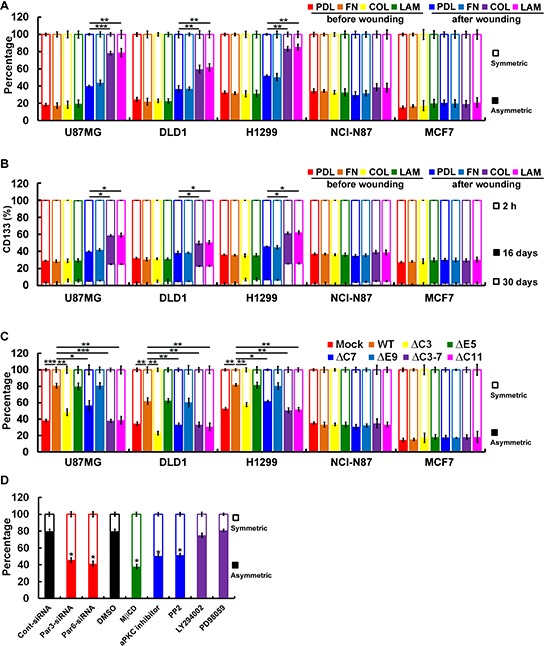
Polarized localization of CD133/integrins in migrating cells contributes to ACD **A.** Subconfluent BrdU-labeled CD133^+^ cells were replated on poly-d-lysine (PDL)-, fibronectin (FN)-, collagen (COL)- or laminin (LAM)-coated dishes and allowed to adhere for 24 h. Wounding was performed by scraping with an 8-channel pipette (with 0.1- to 2-μl tips) several times across the dish. After another 24 h, BrdU asymmetry or symmetry was quantified. **B.** Subconfluent CD133^+^ cells were replated on coated dishes as described above and allowed to adhere for 24 h. Eight (16 days) or 15 (30 days) passages after wounding, the percentage of cells expressing CD133 was determined with flow cytometry. **C.** BrdU asymmetry or symmetry was quantified as described in A by CD133^−^/CD133-HA (WT or mutants) cells. **D.** BrdU-labeled U87MG/CD133^+^cells were cultured on collagen-coated dishes and pretreated with inhibitors as in 5C. BrdU asymmetry or symmetry was quantified 24 h post-wounding. Data were derived from three independent experiments and are presented as the mean ± S.D. *P < 0.05; **P < 0.01; ***P < 0.005 (*t*-test).

Because the mode of cell division in CSCs is critically important, we used pulse-chase BrdU labeling and paired-cell assays to investigate cell division in cancer cells [26]. To determine whether ACD is CD133/integrin dependent, we chased BrdU-labeled CD133^+^ cells cultured on ECM-coated dishes before/after wounding. The frequency of ACD in U87MG, DLD1 and H1299 cells cultured on collagen- or laminin-coated dishes after wounding ranged from about 65% to 85%, which was higher than the ACD frequencies in NCI-N87 (~40–50%) and MCF7 (~20–40%) cells cultured on poly-d-lysine- or fibronectin-coated dishes after wounding (Figure 6A). When 2 weeks of pulsing was followed by a chase period of 0–30 days, ~25% of U87MG, DLD1, and H1299 cells cultured on collagen- or laminin-coated dishes after wounding retained CD133^+^ (Figure 6B). The percentage of CD133^+^ NCI-N87 and MCF7 cells cultured on poly-d-lysine- or fibronectin-coated dishes after wounding began to decline within 16 days during the chase period and had fallen to 1–2% after 30 days (Figure 6B). These results suggest that CD133 is distributed symmetrically in cells cultured on poly-d-lysine- or fibronectin-coated dishes and that induction of differentiation generates CD133^−^ cells. CD133-mediated collagen/laminin-triggered signaling maintained the CSC population in U87MG, DLD1 and H1299 cells. These results were confirmed by the effect of domain mapping CD133 on ACD. Cells expressing CD133ΔC3, CD133ΔC7, CD133ΔC3-7 or CD133ΔC11 did not show a higher frequency of ACD (Figure 6C). Of the inhibitors tested, a lipid raft-destabilizing drug (MβCD), an aPKC inhibitor (Gö6983), a Src inhibitor (PP2) and siRNA targeting Par3 and Par6 disrupted ACD (Figure 6D). By contrast, neither PI3K (LY294002) nor MAPK (PD98059) inhibitors had any effect.

### Polarized CD133-mediated signaling that mediates the noncanonical Wnt pathway is dependent on the CD133/Src/Akt/GSK3β/ β-catenin axis

**Figure 7:**
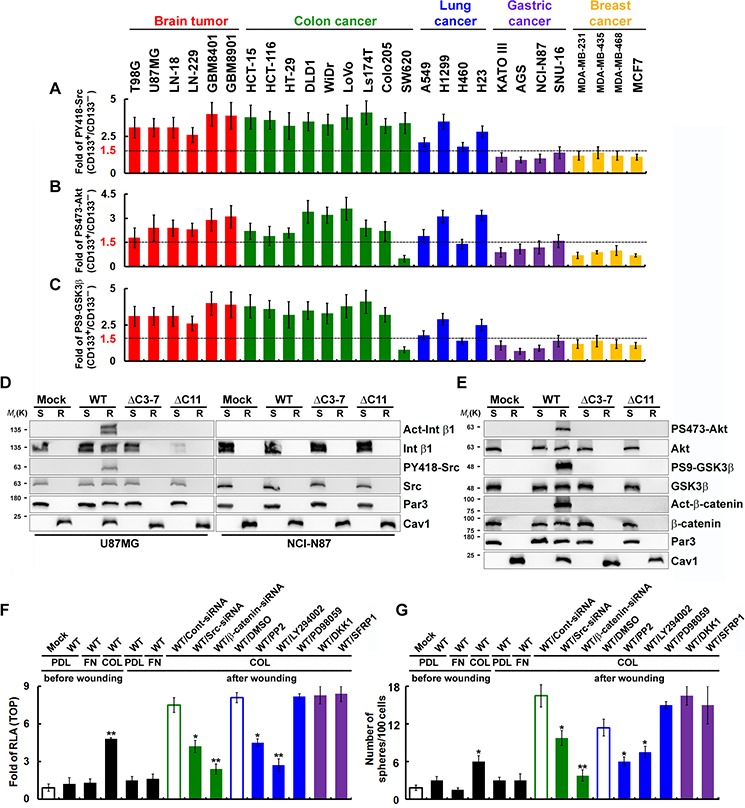
Polarized CD133-mediated signaling through the noncanonical Wnt pathway is dependent on the CD133/Src/Akt/GSK3β/β-catenin axis **A–C.** Cells were fractionated using FACS into CD133^+^ and CD133^−^ fractions. Twenty-four hours post-wounding, cells were incubated with isotype IgG (control) or antibody specifically recognizing PY418-Src (in A), PS473-Akt (in B), or PS9-GSK3β (in C) they labeled with Alexa Fluor 488-conjugated secondary antibody. Fluorescence intensity was determined using flow cytometry, and the SFI was calculated as the ratio of the mean fluorescence obtained with the specific antibody to that obtained with isotype IgG. The CD133^+^/CD133^−^ SFI ratio was then calculated. **D** and **E.** CD133^−^/CD133-HA (WT or mutants) cells cultured on collagen-coated dishes were lysed in cold Triton X-100 buffer 24 h post-wounding. The Triton X-100-insoluble raft (R) and -soluble fractions (S) were analyzed by immunoblotting for the indicated proteins. **F** and **G.** U87MG/CD133^−^/CD133-HA (WT) cells cultured on poly-d-lysine (PDL)-, fibronectin (FN)- or collagen (COL)-coated dishes were pretreated with an inhibitor of Src (PP2), PI3K (LY294002) or MAPK (PD98059); the canonical Wnt pathway antagonist DKK1 or SFRP1; or siRNA targeting Src or β-catenin. TOPflash luciferase reporter (in F) and self-renewal capability (sphere-forming cells) assays (in G) were performed 24 h post-wounding. RLA, relative luciferase activity. Data in (A-C and F-G) were derived from three independent experiments and are presented as the mean ± S.D. *P < 0.05; **P < 0.01 (*t*-test).

Using FACS with monoclonal antibodies that specifically recognized Src-PY418, Akt-PS473 and GSK3β-PS9, we observed robust activation of Src, Akt and GSK3β in CD133^+^ U87MG, DLD1 and H1299 cells, but these kinases were not activated in CD133^+^ NCI-N87 and MCF7 cells (Figure 7A–7C). To further define the regions of CD133 involved in activation of integrin, the effects of Src, Akt and GSK3β were examined in cells expression each of four extracellular- and intracellular-domain CD133 deletion mutants. After wounding, the CD133/integrin/Src/Akt/GSK3βaxis was activated in U87MG cells but not NCI-N87 cells (Figure 7D and 7E). CD133ΔC3-7 and CD133ΔC11 failed to activate the abovementioned kinases after wounding. To determine whether CD133/integrin polarization (in cells cultured on collagen- or laminin-coated dishes) and intact signaling in the CD133/integrin/Src/Akt/GSK3βaxis are required for β-catenin-dependent transcriptional activity, cells were pretreated with inhibitors of Src (PP2), PI3K (LY294002) or MAPK (PD98059), with the canonical Wnt pathway antagonist DKK1 or SFRP1, or with siRNA targeting Src or β-catenin. Pretreatment with inhibitors that perturb the CD133/integrin/Src/Akt/GSK3βaxis abolished CD133-mediated β-catenin-dependent transcriptional activity (Figure 7F) and the CSC phenotype (Figure 7G), whereas the canonical Wnt antagonists, DKK1 and SFRP1, did not. This suggests polarized CD133/integrin-evoked Src activation (in cells cultured on collagen- or laminin-coated dishes) enhanced phosphorylation of GSK3β and its potential upstream regulator Akt as well as the subsequent increase and nuclear translocation of β-catenin.

### Polarized cell migration-induced CD133/integrin/Src/Akt/GSK3β/β-catenin axis is required for maintenance of CSC properties

**Figure 8:**
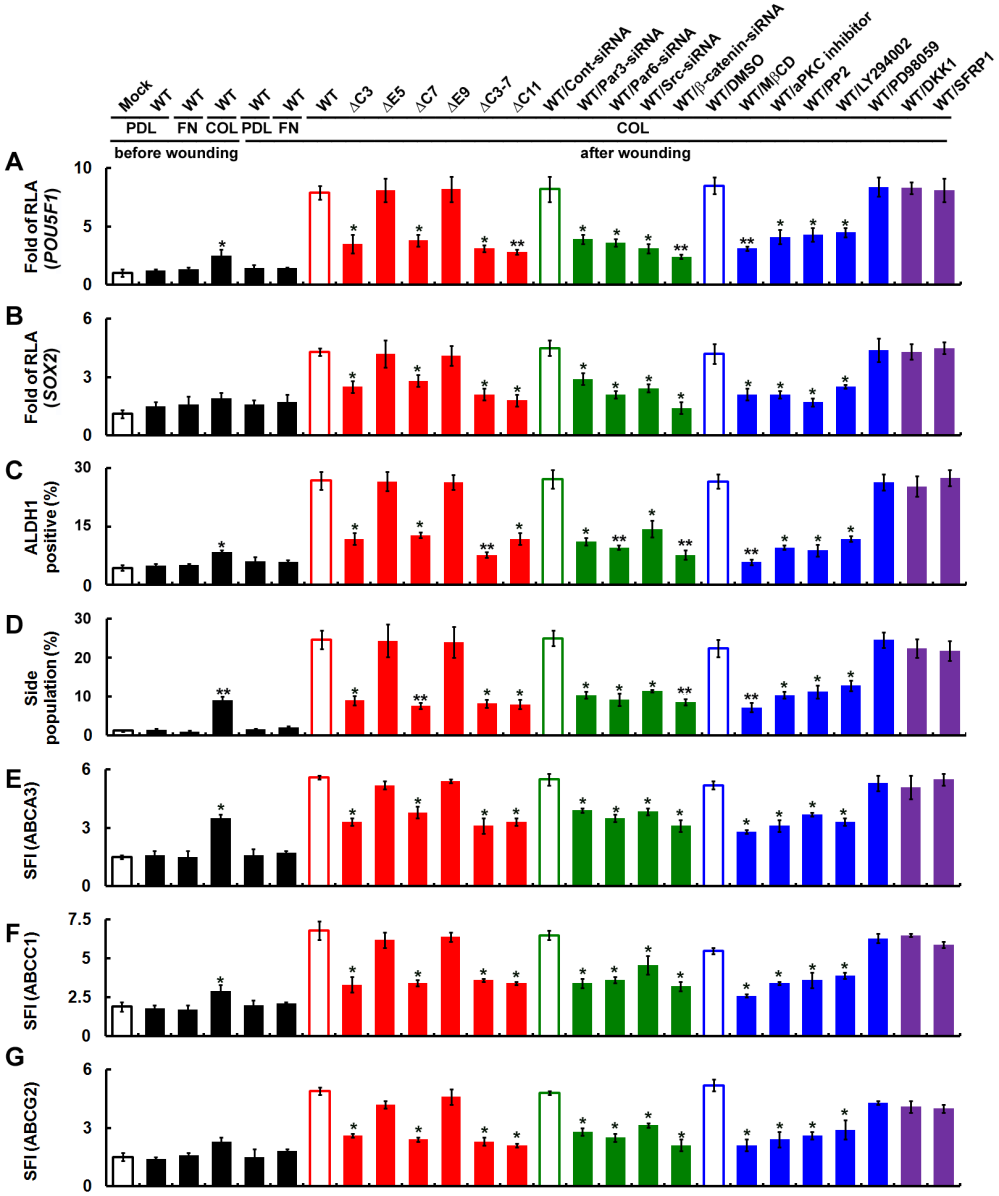
Polarized cell migration induces CD133/integrin/Src/Akt/GSK3β/β-catenin signaling required for maintenance of CSC properties Cells were fractionated with FACS into CD133^+^ and CD133^−^ fractions. U87MG/CD133^−^/CD133-HA (WT or mutants) cells cultured on poly-d-lysine (PDL)-, fibronectin (FN)- or collagen (COL)-coated dishes were pretreated with a lipid raft-destabilizing drug (MβCD) or an inhibitor of aPKC (Gö6983), Src (PP2), PI3K (LY294002) or MAPK (PD98059); the canonical Wnt pathway antagonist DKK1 or SFRP1; or siRNA targeting Par3, Par6, Srcor β-catenin. **A–C.** Twenty-four hours post-wounding, expression of stem cell-related genes was assessed using reporter assays with the *POU5F1* (in A) or *SOX2* (in B) promoter and FACS (ALDH1; in C). RLA, relative luciferase activity. **D.** Cells were stained with Hoechst 33342 24 h post-wounding, and the percentage of SP cells was counted. **E–G.** Twenty-four hours post-wounding, cells were incubated with isotype IgG (control) or antibody specifically recognizing ABC transporter family members (ABCA3 in E, ABCC1 in F, and ABCG2 in G) followed by labeling with Alexa Fluor 488-conjugated secondary antibody. Fluorescence intensity was determined with flow cytometry. The SFI was calculated as in Figure 7. Data were derived from three independent experiments and are presented as the mean ± S.D. *P < 0.05; **P < 0.01 (*t*-test).

We hypothesized that CD133 acts to maintain stem-like properties in polarized migrating cells and induces differentiation. Wounding CD133^+^ cells cultured on collagen-coated dishes induced expression of stem cell-related genes (including *Oct4, Sox2* and *ALDH*), whereas wounding Mock and CD133^+^ cells cultured on fibronectin-coated dishes did not (Figure 8A–8C). Likewise, in cells expressing CD133ΔC3, CD133ΔC7, CD133ΔC3-7 or CD133ΔC11, wounding did not increase expression of stem cell-related genes. On the other hand, cells expressing CD133WT, CD133ΔE5 or CD133ΔE9 showed a ~5-15-fold increase in expression of stem cell-related genes. To determine whether signaling in the CD133/integrin/Src/Akt/GSK3β/β-catenin axis is required for maintenance of CSC properties, cells were pretreated with inhibitors as described in Figure 5C. Pretreatment with inhibitors that perturb CD133/integrin polarization or the CD133/integrin/Src/Akt/GSK3β/β-catenin axis abolished the CD133-induced increase in stem cell-related gene expression (Figure 8A–8C) and the maintenance of CSC properties (Figure 8D), but the canonical Wnt antagonists DKK1 and SFRP1 did not.

Using FACS to assess Hoechst dye exclusion and expression of ABC transporter family members, we next asked whether CD133-expressing cancer cells generated SP cells (cells with CSC properties). After wounding, the SP cell fraction was increased among CD133^+^ cells cultured on collagen-coated dishes (Figure 8D), as was the fraction expressing ABC transporter family members (Figure 8E–8G). By contrast, no increases in SP cells or ABC transporter expression were seen in Mock and CD133^+^ cells cultured on fibronectin-coated dishes after wounding. Likewise, cells expressing CD133ΔC3, ΔC7, ΔC3-7 or ΔC11 of did not exclude Hoechst dye (Figure 8D) or show increased expression of ABC transporter family members (Figure 8E–8G). However, cells expressing CD133 WT, ΔE5 or ΔE9 showed a 2- to 6-fold increase in SP cells (Figure 8D) and increased expression of ABCA3, ABCC1 and ABCG2 (Figure 8E–8G). Moreover, pretreatment with inhibitors that perturb CD133/integrin polarization or the CD133/integrin/Src/Akt/GSK3β/β-cateninaxis abolished CD133-induced increases in SP cells and ABC transporter levels, though the canonical Wnt antagonists DKK1 and SFRP1 did not.

### Expression and polarization of CD133 enhances tumorigenesis in experimental animal models

**Figure 9:**
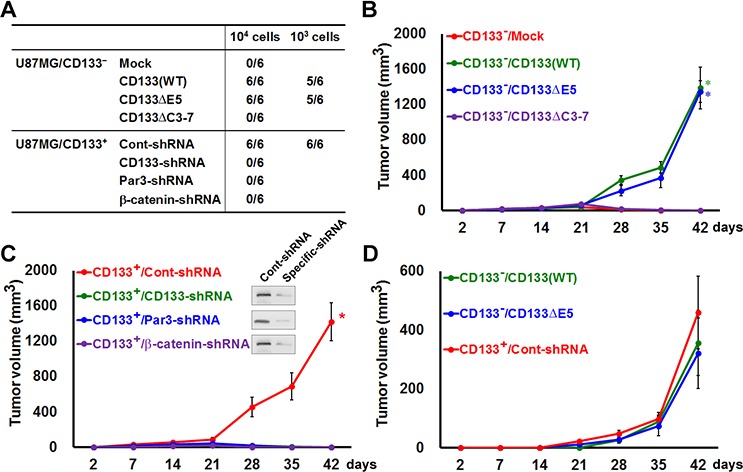
Expression and polarization of CD133 enhances tumorigenesis in an experimental animal model **A.** *In vivo* tumorigenicity was evaluated 6 weeks after subcutaneously injecting mice with 10^3^ or 10^4^ cells in 100 μl of a 1:1 mixture of DMEM/Matrigel. Numbers in the table denote the number of animals with tumors out of the total number of animals in each group. **B–D.** Mice were subcutaneously injected with 10^3^ (in D) or 10^4^ (in B, C) cells as in A. Tumorigenicity was evaluated at 2, 7, 14, 21, 28, 35 and 42 days after transplantation. Tumor volumes are shown. *P < 0.05 (*t*-test).

The *in vivo* tumorigenicity of CD133 was assessed in mice subcutaneously injected with 10^3^ or 10^4^ CD133^+^ that had been cultured on collagen-coated dishes, wounded, and then transfected with siRNA targeting CD133, Par3 or β-catenin, or CD133^−^ cells transfected with WT CD133 or various CD133 mutants. As shown in Figure 9A, CD133^−^/CD133(WT) and CD133^+^/Cont-siRNA cells, which retain CSC properties *in vitro*, were highly tumorigenic *in vivo*. Forty-two days after injection of 10^4^ cells expressing CD133(WT), CD133ΔE5 or CD133^+^/Cont-siRNA six of six animals exhibited tumors with an average volume of 1389 ± 236, 1344 ± 123 and 1423 ± 215 mm^3^, respectively. By contrast, no tumors were observed after injection of cells expressing CD133ΔC3-7 or siRNA targeting CD133, Par3 or β-catenin (Figure 9B–9C). The cells with the highest tumorigenic potential expressed CD133(WT), CD133ΔE5 and CD133^+^/Cont-siRNA [five (356 ± 177), five (321 ± 159) and six (459 ± 123) of six animals, respectively, injected with 10^3^ cells formed tumors] (Figure 9D). Mice injected with cells negative for these proteins did not develop tumors.

## DISCUSSION

CD133 often localizes at plasma membrane protrusions and may influence cell polarity and migration [27]. Our wounding assay revealed that cell migration triggers coalescence of lipid rafts on the plasma membrane, resulting in local enrichment of CD133, Par3, Par6 and integrins on the leading edge. The Par3-Par6-aPKC complex has long been known to regulate the distribution of cell fate determinants and the orientation of microtubules [11], thereby controlling ACD. The function of CD133 during cellular polarization is less well-defined [14]. Because the CD133 mutants used in our study failed to induce coalescence of lipid rafts and resulted in a significantly lower incidence of ACD, we hypothesize that CD133 is also an important contributor to the establishment of cell polarity. CD133 congregates at membrane microdomains that are enriched with sphingolipids and involved in signaling pathways [5], and loss of these CD133-rich microdomains during ACD may lead to cell differentiation [6]. These findings suggest that the polarized distribution of CD133 in a migrating cell may direct that cell toward ACD, renewing the CD133^+^ parental cell while giving rise to a differentiated CD133^−^ daughter cell.

We observed that CD133 mediated ACD and sustained CSC properties in brain, colon and lung cancer cells, but not in gastric or breast cancer cells. CD133 has been used to identify CSCs since it was first reported to be enriched in human brain tumor cells exhibiting an enhanced capacity for self-renewal, proliferation and differentiation [28]. Controversies nonetheless abound regarding the suitability of CD133 to serve as a sole marker for cells with stem-like characteristics. For example, a recent review stated that although CSCs can be purified from CD133^+^ subpopulations in lung, colon, liver, pancreas and skin cancers, CD133 appears to be a general marker for the apical or apicolateral membrane and cannot be used alone to isolate stem cells rich in glandular epithelia, such as from the stomach or the breast [29]. Our experiments also leave some doubt as to whether CD133 positivity properly selected CSCs from among the gastric and breast cancer cells used.

In various types of cancer, the presence of CD133^+^ subpopulations correlates with strong resistance to chemotherapy and/or radiotherapy [30]. Clinicopathological studies have also found that CD133 positivity is associated with more aggressive tumor types [31], advanced stage disease [32] and metastasis [33]. Overall, high CD133 levels portend an unfavorable prognosis [32–34], and several publications have linked a decrease in CD133^+^ cells to reduced tumorigenicity [35–37]. For that reason, strategies have been devised to target CD133 as one approach to cancer treatment. The mechanism by which CD133 facilitates maintenance of CSCs and increases tumorigenicity is not fully understood. It has been proposed that molecular events downstream of CD133 activation include PI3K/Akt activation [9], Hedgehog-GLI1 signaling [37] and Src activation [8, 25]. Our present study establishes that a CD133/integrin/Src/Akt/GSK3β/β-catenin axis and noncanonical Wnt signaling are activated in migrating CSCs, which contributes to a rationale for targeting CD133 in cancer treatment.

**Figure 10:**
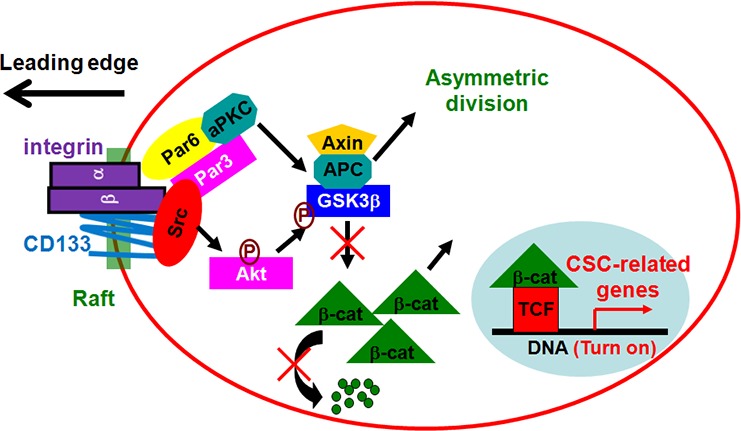
A model for functional cooperation between CD133/integrin and Par complexes in migration In migrating cells, CD133/integrin and Par complexes are recruited to the leading edge of cells to increase Src activity, which enhances CD133/integrin signaling and ACD. In response to directional cues (e.g., integrin activation during migration), polarization of CD133 localization induces lateral reorganization of lipids and membrane-associated proteins. A complex composed of integrin, Src and Par is enriched in lipid rafts, and the assembly and activation of this signaling complex leads to Src activation. CD133-induced Src activation enhances the phosphorylation of GSK3β and its potential upstream regulator, Akt. The subsequent increase and nuclear translocation of β-catenin may be a regulatory switch that increases drug resistance and stemness properties in cancer cells.

Based on our results, we propose the following. In migrating cells, CD133/integrin and Par complexes are recruited to the leading edge of cells to increase Src activity, which enhances CD133/integrin signaling and ACD. In response to directional cues (e.g., integrin activation during migration), polarization of CD133 localization induces lateral reorganization of lipids and membrane-associated proteins. A complex composed of integrin, Src and Par is enriched in lipid rafts, and the assembly and activation of this signaling complex lead to Src activation. CD133-induced Src activation enhances the phosphorylation of GSK3β and its potential upstream regulator, Akt. The subsequent increase and nuclear translocation of β-catenin may be a regulatory switch that increases drug resistance and stemness properties in cancer cells (Figure 10). The present study demonstrates for the first time the contribution made by CD133 to tumor formation and progression in a model where multiple phenotypic cancer cell subpopulations coexist in dynamic equilibrium and where the tumorigenic and metastatic properties of subsets of cells, including CSCs, can be tested concurrently to identify their functional and hierarchical relationships.

## MATERIALS AND METHODS

### Constructs

Wild-type CD133 was purchased from Genediscovery Biotechnology. CD133 mutants with deletions [CD133ΔC3 (deletion of amino acid 130 to 157), CD133ΔE5 (deletion of amino acid 179 to 433), CD133ΔC7 (deletion of amino acid 455 to 486), CD133ΔE9 (deletion of amino acid 508 to 792), CD133ΔC3-7 (deletion of amino acid 130 to 486), and CD133ΔC11 (deletion of amino acid 814 to 865)] were generated through PCR amplification of corresponding cDNA fragments using wild-type CD133 as a template. The luciferase reporter plasmids containing the human *POU5F1* or *SOX2* promoter were gifts from Huck-Hui Ng [38] (Department of Biological Sciences, National University of Singapore, Singapore).

### Antibodies and reagents

The antibodies (Abs) for integrin β1 (B3B11 for detecting total integrin β1 and HUTS-4 for detecting the active form of β1 integrin with immunoblotting) were purchased from Chemicon (Temecula, CA). Abs for caveolin-1, flotillin-2, integrin α2, integrin α3, integrin β6 and Par3 were from Santa Cruz Biotechnology (Santa Cruz, CA). The Ab for Par6 was from Abcam (Cambridge, MA). The Ab for c-Src was from Upstate (Lake Placid, NY). The Abs for CD133, Akt, Akt-pS473, GSK3β, and GSK3β-pS9 were from Cell Signaling Technology (Danvers, MA). The Ab for Src-pY418 was from Biosource (Camarillo, CA). PP2, LY294002, wortmannin, PD98059, and GF109203X were from Calbiochem (San Diego, CA). The Ab for β-catenin and methyl-β-cyclodextrin were from Sigma-Aldrich (St. Louis, MO). The Ab for the active form of β-catenin was from Millipore (Temecula, CA). Alexa 488- and Alexa 594-conjugated anti-mouse or anti-rabbit immunoglobulin G (IgG) were from Molecular Probes (Eugene, OR).

### Human samples and IHC analysis

Sectioned human lung cancer specimens were obtained from GenDiscovery Biotechnology, Inc. Human breast cancer specimens were from Chang-Gung University College of Medicine and Memorial Hospital, Taoyuan. All samples were made anonymous prior to analysis. Studies involving these tissues were approved by the Institutional Review Boards at Chang-Gung University College of Medicine and Memorial Hospital. All staining procedures were performed using a Super Sensitive IHC Detection Systems kit (BioGenex). Counterstaining was performed with hematoxylin. A semi-quantitative method for calculating positive signals was used. Signals were counted in six fields per sample under a light microscope at 400× magnification. The results were evaluated by two independent observers to determine both the percentage of positive cells and the staining intensity, as described [39, 40]. The observers were blinded to the stage of each sample. The IHC score was obtained by multiplying the staining intensity (0 = no expression, 1 = weak expression, 2 = moderate expression, 3 = strong expression and 4 = very strong expression) by the percentage of positive cells (0 = 0–5% expression, 1 = 6–25% expression, 2 = 26–50% expression, 3 = 51–75% expression, and 4 = 76–100% expression) in the field. The maximum possible IHC score was 4 × 4 = 16.

### Immunoblotting

Immunoblotting was performed as described [23]. Images were recorded using a luminescent image analyzer (FUSION SL; Vilber Lourmat, France), and the intensities of the bands were quantitated with densitometry using Bio-1D and Bio-Gene software (Vilber Lourmat).

### Chromatin immunoprecipitation (ChIP)

ChIP was performed as described [39, 41]. Extracted DNA was analyzed using PCR with primers spanning the proximal promoter regions of *ITGA2, ITGA3, ITGA4, ITGA10, ITGA11, ITGB1, ITGB6* or *ITGB8*. Following 30 cycles of amplification, PCR products were run on a 1.5% agarose gel and analyzed with ethidium bromide staining.

### Sphere-forming cultures and self-renewal assays

Spheres were generated as previously described [23]. Briefly, cells were grown in suspension culture (1,000 cells/ml) using ultra-low attachment plates (Corning) and serum-free RPMI (ATCC) supplemented with B27 (Invitrogen), 20 ng/ml EGF and 10 ng/ml bFGF (BD Biosciences). Spheres with a diameter > 30 μm were then counted. For serial passages (self-renewal capability assays), spheres were harvested and dissociated to single cells with trypsin, after which the dissociated cells were plated in a 96-well plate (diluted to 1 cell/well in an ultra-low attachment plate) and cultured for 12 days. The spheres were then counted again. The individual spheres were found to be derived from single cells [42].

### Identification and isolation of side-population (SP) cells

SP cells were determined as described [23]. Cells were suspended in prewarmed RPMI containing 2% FBS and 10 mM HEPES, and were stained with 5 μg/ml Hoechst 33342 dye (Molecular Probes) for 90 min at 37°C with or without 100 μM reserpine, which is an inhibitor of some ATP-binding cassette transporters. Cells were then washed and resuspended in HBSS containing 2% FBS and 10 mM HEPES. Before cell sorting, 0.25 μg/ml 7-AAD (Sigma) was added to exclude nonviable cells. The concentration of Hoechst 33342 and the incubation times were initially identified using samples that provided the highest frequency of SP cells with the lowest cytotoxicity determined by 7-AAD staining. SP cells were analyzed on a FACSAria III (BD Biosciences) flow cytometer equipped with 450/20 nm band pass and 670 nm long pass optical filters (Omega Optical).

### Adhesion assay

Adhesion of cells to plates coated with 10 μg/mL fibronectin, 10 μg/mL collagen, 2 μg/mL laminin, 2 mg/mL poly-d-lysine or 1% heat-inactivated bovine serum albumin in PBS was assessed as described [24, 43]. To obtain a reference value of 100% attachment, cells were seeded on plates precoated with 20 μg/mL fibronectin. Cells were incubated for 3 h at 37°C in a humidified incubator followed by immediate fixation. Approximately 90% to 100% of input cells were recovered.

### 5-bromo-2-deoxyuridine (BrdU) pulse-chase and paired-cell assays

BrdU was added to the culture medium at a concentration of 1 μM for 2 weeks to ensure labeling of all cells. During the pulse, the medium was supplemented with fresh BrdU every 72 h, and cell growth was maintained in log phase. The cells were then synchronized using nocodazole overnight, and flow cytometry was used to confirm BrdU incorporation. For BrdU chasing, the cells were washed thoroughly, seeded onto coated coverslips in BrdU-free medium and synchronized using a thymidine-nocodazole-blebbistatin sequence to halt the cell cycle at the second post-BrdU mitosis and paired-cell formation [26]. The paired cells were fixed and permeabilized, after which the cells were sequentially immersed in 1 N HCl followed by 2 N HCl to open the DNA structure. Immediately after the acid washes, the cells were incubated at room temperature in 0.1 M sodium borate (pH 8.0), then washed and incubated overnight with antibodies specific for BrdU or CSC/differentiation markers.

### Animal experiments

The murine studies were conducted in accordance with the Institutional Animal Care and Use Committee (IACUC) guidelines and were approved by the Animal Care and Use Committee of National Tsing Hua University. Severe combined immunodeficient (i.e., SCID) CB17 female mice (6 weeks old) were used, and all experiments were carried out with the approval of the local authorities. For the *in vivo* tumorigenicity assay, mice were injected subcutaneously with 10^3^ or 10^4^ cells, which were resuspended in 100 μL of a mixture of DMEM/Matrigel (1:1). Tumorigenicity was evaluated 6 weeks after transplantation.
