# Correction: Polarized cell migration induces cancer type-specific CD133/integrin/Src/Akt/GSK3β/β-catenin signaling required for maintenance of cancer stem cell properties

**DOI:** 10.18632/oncotarget.11119

**Published:** 2016-08-08

**Authors:** Ying-Jhen Su, Wei-Hsin Lin, Yi-Wen Chang, Kuo-Chen Wei, Chi-Lung Liang, Shin-Cheh Chen, Jia-Lin Lee

Present: Due to an error made during submission of the revised manuscript, Dr. Chi-Jung Liang's name was spelled incorrectly.

Corrected: The correct author list for this manuscript is provided below. Authors sincerely apologize for this oversight.

Original article: Oncotarget. 2015; 6(35): 38029-45. doi: 10.18632/oncotarget.5703.

Ying-Jhen Su^1,*^, Wei-Hsin Lin^1,2,*^, Yi-Wen Chang^1,*^, Kuo-Chen Wei^3^, Chi-Jung Liang^1^, Shin-Cheh Chen^4^ and Jia-Lin Lee^1,5^

^1^ Institute of Molecular and Cellular Biology, National Tsing Hua University, Hsinchu, Taiwan

^2^ Department of Orthopedics, National Taiwan University Hospital Hsin-Chu Branch, Hsinchu, Taiwan

^3^ Department of Neurosurgery, Chang-Gung Memorial Hospital, Linkou, Taiwan

^4^ Department of Surgery, Chang-Gung Memorial Hospital, Linkou, Taiwan

^5^ Department of Medical Science, National Tsing Hua University, Hsinchu, Taiwan

^*^ These authors have contributed equally to this work

